# Robust observer based control for plasma glucose regulation in type 1 diabetes patient using attractive ellipsoid method

**DOI:** 10.1049/iet-syb.2018.5054

**Published:** 2019-03-11

**Authors:** Anirudh Nath, Rajeeb Dey

**Affiliations:** ^1^ Electrical Engineering Department, National Institute of Technology Silchar Assam 788010 India

**Keywords:** medical control systems, observers, uncertain systems, nonlinear control systems, robust control, control system synthesis, linear matrix inequalities, feedback, sugar, closed loop systems, diseases, virtual T1D patients, type 1 diabetes patients, closed‐loop simulations, uncertain conditions, post‐prandial hyperglycemia, designed controller, closed‐loop response, controller gains, linear matrix inequality, controller conditions, T1D patient model, control technique, intra‐patient parametric variation, principal objective, plasma glucose concentration, uncertain nonlinear system, robust observer based output feedback control law, attractive ellipsoid method, plasma glucose regulation

## Abstract

This paper deals with the design of robust observer based output feedback control law for the stabilisation of an uncertain nonlinear system and subsequently apply the developed method for the regulation of plasma glucose concentration in Type 1 diabetes (T1D) patients. The principal objective behind the proposed design is to deal with the issues of intra‐patient parametric variation and non‐availability of all state variables for measurement. The proposed control technique for the T1D patient model is based on the attractive ellipsoid method (AEM). The observer and controller conditions are obtained in terms of linear matrix inequality (LMI), thus allowing to compute easily both the observer and controller gains. The closed‐loop response obtained using the designed controller avoids adverse situations of hypoglycemia and post‐prandial hyperglycemia under uncertain conditions. Further to validate the robustness of the design, closed‐loop simulations of random 200 virtual T1D patients considering parameters within the considered ranges are presented. The results indicate that hypoglycemia and post‐prandial hyperglycemia are significantly reduced in the presence of bounded (±30% ) parametric variability and uncertain exogenous meal disturbance.

## 1 Introduction

Type 1 diabetes (T1D) patients reckon on multiple exogenous insulin infusions since their body is unable to secrete insulin (which is the primary regulator of glucose homeostasis), repercussion in prolonged elevated plasma glucose concentration (PGC). The extreme glucose excursions in both the direction from the euglycemic range of 70–180 mg/dl lead to hyperglycemia (PGC>180 mg/dl) and severe hypoglycemia (PGC<50 mg/dl) [1 ]. Both of these have adverse effects on the health of the T1D patients, that range from long‐term effects (due to hyperglycemia) such as coronary disorder, neuropathy, nephropathy, retinopathy, diabetes ketoacidosis etc to short‐term effect (due to hypoglycemia) that can lead to diabetic coma [2, 3 ].

The artificial pancreas (AP) system is essentially an automatic closed‐loop system for the exogenous insulin delivery via insulin pump as determined by a control algorithm based on the glucose measurements provided by the glucose sensors [4 ]. The issues of inter‐patient variability (parametric variability within a population of T1D patients) and intra‐patient variability (parametric variability within the same T1D patient) emanates out of the existence of high uncertainty existing in the physiological factors, such as insulin sensitivity (IS) and various other factors affecting the glucose‐insulin dynamics [5 ]. This poses a serious hindrance to the practical realisation of the automatic control algorithm that constitutes the core of an AP.

Mathematical models that constitute the core of the model‐based control algorithms can be classified into intravenous and the subcutaneous T1D models, where the glucose measurements and insulin infusions are done intravenously or subcutaneously, respectively # [6 ]. In this paper, well known nonlinear intravenous Bergman's minimal model (BMM) [7 ] is taken into consideration for the design of the control algorithm. The principal reasons behind selecting the BMM are: (i) it models the macroscopic response of the complex glucose‐insulin dynamics via a simple nonlinearity with an acceptable degree of accuracy [8 ], (ii) it has a very high applicability in ‘bed‐side AP’ [9 ] that is crucial for the treatment of T1D patients with diabetes ketoacidosis and in the intensive care unit (ICU) [10 ] and (iii) important physiological factors like, glucose effectiveness, insulin sensitivity and insulin degradation rate can be easily modelled in terms of its parameters [8 ].

The most significant issues in the domain of control of nonlinear systems are: (i) parametric uncertainty and modelling inaccuracy, (ii) presence of immeasurable states [11 ] and (iii) effect of exogenous disturbances. Many biological systems that are governed by nonlinear dynamics require the observers for estimating the immeasurable states by utilising the information regarding the measurable states [12 ]. Due to the absence of insulin sensors in AP systems, the design of an appropriate observer is very crucial. Furthermore, the time‐varying uncertainty in the form of inter‐patient variability and intra‐patient variability, that exist in the nonlinear dynamics of the T1D, along with the impact of exogenous meal disturbance, makes the problem of regulating the PGC quite challenging. [13 ]

The philosophy of this current work is motivated from the work of [14 ] where an output feedback control law based on observer was designed for a nominal nonlinear system. In the current work, the control philosophy of [14 ] is extended by incorporating parametric uncertainty and exogenous disturbance using the Attractive Ellipsoid Method (AEM) [15 ] in a robust framework for the first time.

Several attempts have been made in the recent past towards addressing the problem of state estimation as discussed in [8 ]. The discussion on the state estimation problem in T1D will be restricted to BMM only. The state estimation problem of the glucose‐insulin system can be categorised into two major categories: (i) observer based methods and (ii) Kalman filter (KF) based methods. A Luenberger observer was formulated for the BMM in [16 ] where the external incoming glucose disturbance is taken into consideration. The estimated states were then utilised in a disturbance‐rejection linear quadratic regulator (LQR). Similarly, in [17 ], the observer design was followed by proportional integral derivative (PID) control algorithm. Furthermore, an input‐output feedback linearisation control law based on a nonlinear observer was proposed for BMM and robustness to bounded parametric uncertainty was shown through random numerical simulations [18 ]. But the incorporation of the parametric uncertainty both in the design of observer and control law was not addressed. In [19 ], the BMM is utilised with different variants of KFs, namely, unscented Kalman filter (UKF) and the extended Kalman filter (EKF) in order to estimate the plasma insulin concentration. Another important work on UKF based on the BMM was reported in [20 ] where the endogenous insulin secretion was taken into account. The major difficulties pertaining to the above mentioned works on state‐estimation of the BMM are stated as follows: (i) all the above‐cited state estimation approaches [16 –20 ] do not take the intra‐patient variability (time‐varying parametric uncertainty) explicitly in their design, (ii) the observer design carried out in [16, 17 ] are based on linear/linearised models of T1D that may lead to significant loss of information embedded in the nonlinear characteristics of the BMM, (iii) the state estimation via KF, EKF or UKF require accurate information about the system model and error distribution (which may not be obtained easily) and may lead to approximation errors due to underestimation of state uncertainties [19 ]. In order to address the adduced issues, a robust observer is designed for the nonlinear intravenous BMM in the present work using AEM [15 ] that exploits the linear matrix inequality (LMI) framework.

Most of the model‐based control design for diabetes patients are found to be based on PID control [17 ], model predictive control (MPC) [1 ], fuzzy logic control [21 ], adaptive control [22 ]. The inherent disadvantages of the above control designs are, PID design cannot deal with intra‐patient parametric variability and nonlinearity in the system, MPC has demerits of computational complexity, dealing with time‐delay and the coupling effects, Fuzzy logic control does not consider nonlinear & robust analysis of the system dynamics and are completely dependent on the rules designed by the experts, lastly the adaptive control techniques becomes cumbersome in terms of parameter adaptation law, as the dynamics of subcutaneous models become more complex. In a sequel, a tractable robust controller design method is more suitable for such a bio‐medical system and hence is the focus of the present work. The two major classes of robust control techniques that are applied for blood glucose regulation in T1D models (intravenous and subcutaneous) are, $H_{\infty }$ control [23 –25 ] and different variants of sliding mode control (SMC) [26 –28 ]. The main issues in the $H_{\infty }$ control and the SMC based control are summarised as (i) $H_{\infty }$ controllers are often of high order and fragile in nature, (ii) SMC based techniques suffer from inherent chattering phenomenon that may result in aggressive exogenous insulin infusions leading to hypoglycemia.

In context to the foregoing discussion on the existing methods for regulation of PGC in diabetes patients, a robust observer based output feedback controller for the nonlinear BMM utilising the analytic framework of AEM for a class of Lipschitz nonlinear system in the presence of time‐varying parametric uncertainty (intra‐patient variability) as well as exogenous meal disturbance is proposed for the first time. The theoretical contribution of the current work is the extension of the observer based output feedback stabilisation of nonlinear system which was presented in [14 ] to a robust framework. The main highlights of the current work are:

- A robust observer based on AEM estimates the immeasurable states of the BMM in the presence of ±30% parametric uncertainty and random meal disturbances.
- A robust state feedback control law is proposed for the BMM that provides optimal insulin dosage when model parameters are different for different subjects (intra‐patient variability).
- The observer gain and the controller gain are optimal as they are computed by solving a constrained optimisation problem with LMI conditions, thereby, ensuring analytical and numerical tractability.
- An output feedback control law was designed to (i) stabilise the uncertain nonlinear T1D system in the presence of exogenous meal disturbance and (ii) meet the required control performance by avoiding hypoglycemia.
- Finally, control variability grid analysis (CVGA) of 200 virtual T1D patients under the proposed observer based output feedback control law is performed for the evaluation of the efficacy as well as validation of the reliability of the proposed control technique.

The paper is organised into four major sections. Section 2 presents the mathematical model of the T1D patient, the design of the robust observer and the robust output feedback control law. The simulation results for different scenarios of the patients are presented in Section 3. Section 4 summarises the present work along with the scope of future extension of the work.

## 2 Problem formulation

Since BMM is a well known and popular T1D model that has been extensively studied and analysed in the literature, thus, the physiological intravenous BMM for T1D is presented briefly in the first Subsection.

### 2.1 Mathematical model of T1DM patients

The BMM reported in [22, 26 ] is considered for designing the observer and controller. The state space formulation of the BMM is presented below:

(1) $$\begin{array}{rl} {\dot{x}}_{1}(t) & =-c_{1}(x_{1}(t)-G_{b})-x_{1}(t)x_{2}(t)+d(t)\\ {\dot{x}}_{2}(t) & =-c_{2}x_{2}(t)+c_{3}(x_{3}(t)-I_{b})\\ {\dot{x}}_{3}(t) & =-c_{4}(x_{3}(t)-I_{b})+u(t) \end{array}$$

where $x_{i}(t),i=1,\ldots ,3$ represent the PGC (mg/dl), the delayed insulin action ($\mathrm{mi}n^{-1}$ ), the plasma insulin concentration (mU/L). $G_{b}$ and $I_{b}$ represent the basal (steady‐state) values of PGC and insulin concentration, respectively. The first ordinary differential equation (ODE) models the plasma glucose dynamics which tell us that the PGC is nonlinearly affected by the state $x_{2}(t)$ and the parameter $c_{1}$ ($\mathrm{mi}n^{-1}$ ) denotes the insulin independent glucose utilisation factor (glucose effectiveness) [22 ]. The ratio $c_{3}/c_{2}$ (L/(min×mU) ) stands for the insulin sensitivity in the second ODE that accounts for the delayed action of insulin on the PGC. The third ODE explains the insulin kinetics with the control input (external insulin infusion) u(t) appearing externally to it. The parameter $c_{4}$ ($\mathrm{mi}n^{-1}$ ) stands for the insulin degradation rate [26 ]. The meal disturbance d(t) affects the PGC externally following a meal ingestion where $c_{5}$ ($\mathrm{mi}n^{-1}$ ) is the rate of appearance of meal disturbance in the plasma glucose compartment.

(2) $$\dot{d}(t)=-c_{5}d(t)$$

#### 2.1.1 Representation in deviated states

Following the method in [22 ] the system in (1 ) can be represented in deviated states as described below.

(3) $$\begin{array}{rl} {\dot{x}}_{1_{d}}(t) & =-c_{1}x_{1_{d}}(t)-(x_{1_{d}}(t)+G_{b})x_{2_{d}}(t)+d(t)\\ {\dot{x}}_{2_{d}}(t) & =-c_{2}x_{2_{d}}(t)+c_{3}x_{3_{d}}(t)\\ {\dot{x}}_{3_{d}}(t) & =-c_{4}x_{3_{d}}(t)+u(t) \end{array}$$

where, $x_{d}(t)={\left[\begin{array}{ccc} x_{1_{d}}(t) & x_{2_{d}}(t) & x_{3_{d}}(t) \end{array}\right]}^{T}$ is the deviated state about the equilibrium point $x_{0}={\left[\begin{array}{ccc} x_{1_{0}} & x_{2_{0}} & x_{3_{0}} \end{array}\right]}^{T}={\left[\begin{array}{ccc} G_{b} & 0 & I_{b} \end{array}\right]}^{T}={\left[\begin{array}{ccc} 80 & 0 & 7 \end{array}\right]}^{T}$ of the system (1 ). The above system can be rewritten in compact form as below,

(4) $${\dot{x}}_{d}(t)=Ax_{d}(t)+Bu(t)+\phi (x_{d}(t))+Dd(t)$$

(5) $$y\left(t\right)=Cx_{d}(t)=x_{1_{d}}(t)$$

where

$$A=\left[\begin{array}{ccc} -c_{1} & G_{b} & 0\\ 0 & -c_{2} & c_{3}\\ 0 & 0 & -c_{4} \end{array}\right],$$

$\phi (x_{d})={\left[\begin{array}{cccc} -x_{1_{d}}(t) & x_{2_{d}}(t) & 0 & 0 \end{array}\right]}^{T}$, and $B={\left[\begin{array}{ccc} 0 & 0 & 1 \end{array}\right]}^{T}$, $D={\left[\begin{array}{ccc} 1 & 0 & 0 \end{array}\right]}^{T}$, $C=\left[\begin{array}{ccc} 1 & 0 & 0 \end{array}\right]$, $x_{d}\in {ℜ}^{n}$, $u(t)\in {ℜ}^{m}$, $y(t)\in {ℜ}^{p}$, with n=3, m=1 and p=1 . The modern Artificial Pancreas System (APS) is equipped with glucose measuring devices that provide continuous glucose measurements periodically that is crucial for the controller design.

The pair (*A*, *B* ) and (*A*, *C* ) are found to be controllable and observable for the considered model.

#### 2.1.2 Uncertain model

Considering the range of parameters placed in Table 1, system (3 ) can now be represented as an uncertain system,

(6) $${\dot{x}}_{d}(t)=\left(A+\Delta A(t)\right)x_{d}(t)+Bu(t)+\phi (x_{d}(t))+Dd(t)$$

where, *A* is the known system matrix containing the parameters, $c_{i},i=1,\ldots ,4$ referred as nominal part, ΔA(t) represents the uncertain part of the system matrix, where the elements vary within a plausible range of $c_{i},i=1,\ldots ,4$ as mentioned in Table 1. For physiological models it is valid to assume that the states and parameters of (6 ) are all bounded for specific patient (or subject). The worst‐case norm for the states, disturbance and the uncertain matrix are considered as,

(7) $$∥x_{d}\left(t\right)∥\leq ∥x_{d_{\max}}∥=X_{+},∥d(t)∥\leq D_{+},∥\Delta A\left(t\right)∥\leq \delta$$

where $X_{+}>0,D_{+}>0\;\mathrm{and}\;\delta >0$ are known apriori and $x_{d_{\max}}$ represents the maximum deviation of $x_{d}(t)$ . The elements of the uncertain matrix, $\Delta A\left(t\right)$ vary within an interval as provided in Table 1. $∥\Delta A\left(t\right)∥$ is the worst case norm of the uncertain matrix calculated using the parametric variations as given in Table 1.

**Table 1:** Nominal and range of parameters for the model (1 ) [22 ]

Parameters	Values	Range
*c*_1_	0	—
*c*_2_	0.015	[0.0105, 0.0195]
*c*_3_	2 × 10^−6^	[1.4 × 10^−6^, 2.6 × 10^−6^ ]
*c*_4_	0.2	[0.14, 0.26]
*c*_5_	0.05	[0.045, 0.055]

Deviated states	Equilibrium point	Range
$x_{1_{d}}$	0	[−80 400]
$x_{2_{d}}$	0	[0 0.01]
$x_{3_{d}}$	0	[−7 40]

Disturbance	Equilibrium point	Initial value
d(*t* )	0	[0 10]

**Note:** The minimal model parameter $c_{1}$ is negligible in T1DM patients and hence the value is considered as $c_{1}=0$ in this current work [26 ].

**Fig 1:**
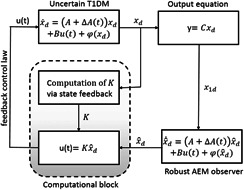
Block diagram representation of the proposed observer based output feedback control technique

The design philosophy of the current research work is illustrated in Fig. 1 where it is made clear that the robust control law based on AEM utilises the estimated state information provided by the robust AEM observer. As discussed in Sub‐section 2.2, the formulation of the problem can be apportioned into three stages: (i) Stage 1: design of the robust AEM observer where an optimal robust observer gain, *L* ensures the convergence of the state estimation error, (ii) Stage 2: compute state feedback gain with parametric uncertainties ensuring the convergence of the states and finally (iii) Stage 3: design of the output feedback control law using the estimated state from the observer and the state feedback gain from the controller.

The potential advantage of this method is that it does not require to test the controllability and observability of the nonlinear system, observer and controller gains can be computed separately and more importantly the implementation is output feedback law and not the state feedback law.

### 2.2 Design of robust AEM observer

In this subsection, a robust observer is designed for a class of Lipschitz nonlinear uncertain system (6 ) adopting the AEM [15 ] in an LMI framework. This design guarantees the boundedness of the state estimation errors to asymptotically converge within a convex region referred as attractive ellipsoid in the presence of parametric uncertainty.

Following definition and Lemmas will be useful to design an observer for the system (6 ) in an LMI framework.

[15 ] Let us consider an ellipsoid $E(P_{\mathrm{attr}})$ . It is said to be an attractive ellipsoid for the trajectory $e_{o}(t),t>0$ if it tends asymptotically inside this ellipse. Mathematically, it can be expressed as: ${\lim\sup}_{t\to \infty }e_{o}^{T}\left(t\right)P_{\mathrm{attr}}e_{o}\left(t\right)\leq 1$.

A nonlinear function $\Phi (x_{d})$ is called Lipschitz function, if it satisfies,

(8) $$∥\phi (x_{d}(t))-\phi ({\hat{x}}_{d}(t))∥\leq L_{\phi }∥x_{d}(t)-{\hat{x}}_{d}(t)∥$$

for any $(x_{d}(t),{\hat{x}}_{d}(t))\in {ℜ}^{n}$ and $L_{\phi }>0$ in (8 ) is called Lipschitz constant.

*(Schur compliement lemma:):* [29 ] For any matrices *G*, *H* and *I*, $\left[\begin{array}{cc} G & H\\ H^{T} & R \end{array}\right]$ is equivalent to

(9) $$I<0,\;G-HI^{-1}H^{T}<0$$

The objective is to design a robust observer that provides bounded estimated state trajectories of the system (6 ) in presence of uncertain parameters and disturbance. Further, the design ensures that all the trajectories remain within the attractive ellipsoid of ‘minimal size’.

Let us consider a Luenberger like observer for the uncertain system (6 ),

(10) $${\dot{\hat{x}}}_{d}\left(t\right)=A{\hat{x}}_{d}\left(t\right)+Bu\left(t\right)+\phi \left({\hat{x}}_{d}\left(t\right)\right)+L\left[y\left(t\right)-C{\hat{x}}_{d}\left(t\right)\right]$$

where *L* is the observer gain matrix, $y(t)=Cx_{d}(t)=x_{1_{d}}(t)$ is output of the uncertain T1DM model (6 ) with $C=\left[\begin{array}{ccc} 1 & 0 & 0 \end{array}\right]$ . Let us define the error of the state estimation as

(11) $$e_{o}\left(t\right):=x_{d}\left(t\right)-{\hat{x}}_{d}\left(t\right)$$

Differentiating the error signal and substituting the values from (6 ) and (10 ) one can get,

(12) $$\begin{array}{rl} {\dot{e}}_{o}\left(t\right) & ={\dot{x}}_{d}\left(t\right)-{\dot{\hat{x}}}_{d}\left(t\right)=\left[A-LC\right]e_{o}\left(t\right)\\ & \;+\Delta A\left(t\right)x_{d}\left(t\right)+\Delta \phi \left(t\right)+Dd(t) \end{array}$$

where, $\Delta \phi \left(t\right):=\phi \left(x\left(t\right)\right)-\phi \left(\hat{x}\left(t\right)\right)$ satisfying the lemma in (8 ). The observer design is presented in the form of following theorem next.

For the system (6 ) satisfying the uncertainty bounds in (7 ) and the Lipschitz condition (8 ), if there exists a matrix $P=P^{T}>0$, corresponding observer gain matrix *L* and positive constants α>0,ε>0 such that,

(13) $$\tilde{W}\left(P,L|\alpha ,\varepsilon \right)=\left[\begin{array}{cc} \Lambda_{11} & P\\ P & -\varepsilon I_{n\times n} \end{array}\right]<0$$

where $\Lambda_{11}=\Lambda +\varepsilon L_{\phi }^{2}I_{n\times n}$, *L* being the observer gain matrix, $\Lambda =P\left(A+\left(\alpha /2\right)I_{n\times n}\right)+{\left(A+\left(\alpha /2\right)I_{n\times n}\right)}^{T}P-YC-C^{T}Y^{T}$, Y=PL, then one can guarantee,

(14) $$\dot{V}\left(e_{o}\left(t\right)\right)\leq -\alpha V\left(e_{o}\left(t\right)\right)+\varepsilon (\delta^{2}X_{+}^{2}+D_{+}^{2})$$

which implies,

(15) $$V\left(e_{o}\left(t\right)\right)\leq V\left(e_{o}\left(0\right)\right)e^{-\alpha t}+\frac{\varepsilon (\delta^{2}X_{+}^{2}+D_{+}^{2})}{\alpha }\left(1-e^{-\alpha t}\right)$$

and subsequently (15 ) at infinite time leads to,

(16) $$\lim\underset{t\to \infty }{\sup}V\left(e_{o}\left(t\right)\right)\leq \frac{\varepsilon (\delta^{2}X_{+}^{2}+D_{+}^{2})}{\alpha }.$$

Further (16 ) can be equivalently written as,

(17) $$\lim\underset{t\to \infty }{\sup}e_{o}^{T}\left(t\right)\left[\frac{\alpha }{\varepsilon (\delta^{2}X_{+}^{2}+D_{+}^{2})}P\right]e_{o}\left(t\right)\leq 1$$

where $P_{\mathrm{attr}}=\left(\alpha /\left(\varepsilon (\delta^{2}X_{+}^{2}+D_{+}^{2})\right)\right)P$, indicating that the estimation error converges to an attractive ellipsoid as defined in Definition 1.

Let us consider a positive definite storage function,

(18) $$V\left(e_{o}(t)\right):=e_{o}(t){\;}^{T}Pe_{o}(t)$$

where $P=P^{T}>0$ . Differentiating on both sides

(19) $$\dot{V}\left(e_{o}\left(t\right)\right)=2e_{o}^{⊺}\left(t\right)P{\dot{e}}_{o}\left(t\right).$$

Substituting (12 ) in (19 ) and expressing it in the quadratic form one can write,

(20) $$\begin{array}{rl} \dot{V}\left(e_{o}^{T}\left(t\right)\right) & =2e_{o}^{⊺}\left(t\right)P([\underset{A_{o}}{\underbrace{A-LC}}]e_{o}\left(t\right))\\ & \;+2e_{o}^{⊺}\left(t\right)P(\underset{\xi \left(t\right)}{\underbrace{\Delta A\left(t\right)x\left(t\right)+\Delta \phi \left(t\right)+Dd\left(t\right)}}) \end{array}$$

□

Using Lemma 1 in (8 ) and adding and subtracting $\varepsilon I_{n\times n}$ and $\alpha V(e_{o})$ on the left side of the above expression (20 ), one can obtain,

$$\begin{array}{rl} \dot{V}\left(e_{o}\left(t\right)\right) & ={\left(\begin{array}{c} e_{o}\left(t\right)\\ \xi \left(t\right) \end{array}\right)}^{T}\left[\begin{array}{cc} \Lambda & P\\ P & -\varepsilon I_{n\times n} \end{array}\right]\left(\begin{array}{c} e_{o}\left(t\right)\\ \xi \left(t\right) \end{array}\right)\\ & \;+\varepsilon {∥\xi \left(t\right)∥}^{2}-\alpha V\left(e_{o}\left(t\right)\right) \end{array}$$

where $\Lambda =PA_{o}+A_{o}^{T}P+\alpha P$ .

(21) $$\begin{array}{rl} \dot{V}\left(e_{o}\left(t\right)\right) & \leq {\left(\begin{array}{c} e_{o}\left(t\right)\\ \xi \left(t\right) \end{array}\right)}^{T}\left[\begin{array}{cc} \Lambda & P\\ P & -\varepsilon I_{n\times n} \end{array}\right]\left(\begin{array}{c} e_{o}\left(t\right)\\ \xi \left(t\right) \end{array}\right)\\ & +\varepsilon ({∥\Delta A(t)∥}^{2}{∥x_{d}(t)∥}^{2}+{∥d(t)∥}^{2}\\ & +L_{\phi }^{2}{∥e_{o}(t)∥}^{2})-\alpha V\left(e_{o}\left(t\right)\right) \end{array}$$

Using the bounds in (7 ) and the Lipschitz condition in Lemma (8 ) and applying in (21 )

(22) $$\begin{array}{rl} \dot{V}\left(e_{o}\left(t\right)\right)\leq & {\left(\begin{array}{c} e_{o}\left(t\right)\\ \xi \left(t\right) \end{array}\right)}^{T}\left[\begin{array}{cc} \Lambda_{11} & P\\ P & -\varepsilon I_{n\times n} \end{array}\right]\left(\begin{array}{c} e_{o}\left(t\right)\\ \xi \left(t\right) \end{array}\right)\\ + & \varepsilon (\delta^{2}X_{+}^{2}+D_{+}^{2})-\alpha V\left(e_{o}\left(t\right)\right) \end{array}$$

where $\Lambda_{11}=\Lambda +\varepsilon L_{\phi }^{2}I_{n\times n}$ . Notice that the matrix inequality (23 ) can be represented as LMI by expanding the first element of $\tilde{W}\left(P,L|\alpha ,\varepsilon \right)$, i.e. $\Lambda_{11}$ and then defining new variable Y=PL as follows:

(23) $$\tilde{W}\left(P,L|\alpha ,\varepsilon \right)=\left[\begin{array}{cc} \Lambda_{11} & P\\ P & -\varepsilon I_{n\times n} \end{array}\right]<0$$

such that the observer gain matrix can be obtained as:

(24) $$L=P^{-1}Y$$

It is to be worth mentioning at this stage that, minimal size of the ellipsoid is computed using certain optimisation criterion such that the error between actual state and estimated states are minimal. The result is presented in the form of corollary next.

To obtain the optimal observer gain matrix $L^{*}$, the trace of $P_{\mathrm{att}r_{c}}$ is minimised such that the estimated states of the observer (10 ) converges to an attractive ellipsoid of ‘minimal’ size. The mathematical formulation of the statement is described below,

(25) $$\frac{\alpha }{\varepsilon (\delta^{2}X_{+}^{2}+D_{+}^{2})}\mathrm{tr}P\to \underset{P>0,L,\alpha >0,\varepsilon >0}{\lim\sup}$$

satisfying,

(26) $$\tilde{W}\left(P_{\mathrm{attr}},L^{*}|\alpha ,\varepsilon \right)<0$$

and the optimal observer gain matrix, $L^{*}$ is computed as

(27) $$L^{*}=P{{}_{\mathrm{attr}}^{*}}^{-1}Y$$

### 2.3 Design of robust AEM state feedback control law

This sub‐section deals with the design of robust state feedback control law for the uncertain system in (6 ) based on AEM, with the assumption that all the states are available for measurement. The design philosophy behind this technique is mainly motivated from the robust controller design for nonlinear system [14 ] based on the AEM. The structure of the control law is

(28) $$u(t)=Kx_{d}(t)$$

The closed‐loop system under the state feedback control law u(t) applied to (6 ) leads to

(29) $$\begin{array}{rl} {\dot{x}}_{d}(t) & =\left(A+\Delta A(t)\right)x_{d}(t)+BKx_{d}(t)+\phi (x_{d}(t))+Dd(t)\\ & =(\underset{A_{c}}{\underbrace{A+BK}})x_{d}(t)+\underset{\xi (t)}{\underbrace{\Delta A(t)x_{d}(t)+\phi (x_{d}(t))+Dd(t)}} \end{array}$$

For the system (6 ) satisfying the uncertainty bounds in (7 ) and the Lipschitz condition (8 ), if there exists a matrix $P_{c}=P_{c}^{T}>0$, corresponding feedback controller gain matrix *K* and positive constants $\alpha_{c}>0,\varepsilon_{c}>0$ such that,

(30) $${\tilde{W}}_{c}\left(P_{c},K|\alpha_{c},\varepsilon_{c}\right)=\left[\begin{array}{cc} \Lambda_{11c} & P_{c}\\ P_{c} & -\varepsilon_{c}I_{n\times n} \end{array}\right]<0$$

where $\Lambda_{11c}=\Lambda_{c}+\varepsilon_{c}L_{\phi }^{2}I_{n\times n}$, *K* being the controller gain matrix, $\Lambda_{c}=\left(A+\left(\alpha_{c}/2\right)I_{n\times n}\right)X+X{\left(A+\left(\alpha_{c}/2\right)I_{n\times n}\right)}^{T}+BY_{c}+Y_{c}^{T}B^{T}$, $Y_{c}=KP_{c}^{-1}$, $X=P_{c}^{-1}$ then one can guarantee,

(31) $${\dot{V}}_{c}\left(x_{d}\left(t\right)\right)\leq -\alpha_{c}V_{c}\left(x_{d}\left(t\right)\right)+\varepsilon_{c}(\delta^{2}X_{+}^{2}+D_{+}^{2})$$

which implies,

(32) $$V_{c}\left(x_{d}\left(t\right)\right)\leq V_{c}\left(x_{d}\left(0\right)\right)e^{-\alpha t}+\frac{\varepsilon_{c}(\delta^{2}X_{+}^{2}+D_{+}^{2})}{\alpha }\left(1-e^{-\alpha t}\right)$$

and subsequently (32 ) at infinite time leads to,

(33) $$\lim\underset{t\to \infty }{\sup}V_{c}\left(x_{d}\left(t\right)\right)\leq \frac{\varepsilon_{c}(\delta^{2}X_{+}^{2}+D_{+}^{2})}{\alpha }.$$

Further (33 ) can be equivalently written as,

(34) $$\lim\underset{t\to \infty }{\sup}x_{d}^{T}\left(t\right)\left[\frac{\alpha }{\varepsilon (\delta^{2}X_{+}^{2}+D_{+}^{2})}P_{c}\right]x_{d}\left(t\right)\leq 1$$

where $P_{\mathrm{attr}c}=\frac{\alpha }{\varepsilon (\delta^{2}X_{+}^{2}+D_{+}^{2})}P_{c}$, indicating that the state $x_{d}(t)$ converges to an attractive ellipsoid as defined in Definition 1.

Let us consider a positive definite quadratic storage function

(35) $$V_{c}\left(x_{d}(t)\right):=x_{d}(t){\;}^{T}P_{c}x_{d}(t),P_{c}=P_{c}^{T}>0$$

Differentiating (35 ) followed by substituting (47 ) one can write,

(36) $${\dot{V}}_{c}(x_{d}^{T}\left(t\right))=2x_{d}^{⊺}\left(t\right)P_{c}(A_{c}+\xi (t))$$

Expressing (36 ) in quadratic form and applying following steps: (i) adding and subtracting $\varepsilon_{c}\xi^{2}(t)$ and $\alpha_{c}V_{c}(x_{d}(t))$ on the right side of (36 ), (ii) then using (8 ) and (7 ) and (iii) introducing a new variavle $\Lambda_{o}=P_{c}A_{c}+A_{c}^{⊺}P+\alpha_{c}P_{c}+\varepsilon_{c}L_{\phi }^{2}I_{n\times n}$, one can find

(37) $$\begin{array}{rl} {\dot{V}}_{c}\left(x_{d}\left(t\right)\right) & ={\left(\begin{array}{c} x_{d}\left(t\right)\\ \xi \left(t\right) \end{array}\right)}^{T}W_{c}(P_{c},K|\alpha_{c},\varepsilon_{c})\left(\begin{array}{c} x_{d}\left(t\right)\\ \xi \left(t\right) \end{array}\right)\\ & \;+\varepsilon_{c}(\delta^{2}X_{+}^{2}+D_{+}^{2})-\alpha_{c}V_{c}\left(x_{d}\left(t\right)\right) \end{array}$$

where $W_{c}(P_{c},K|\alpha_{c},\varepsilon_{c})=\left[\begin{array}{cc} \Lambda_{o} & P_{c}\\ P_{c} & -\varepsilon_{c}I_{n\times n} \end{array}\right]$ . Notice that $W_{c}(P_{c},K|\alpha_{c},\varepsilon_{c})$ is a BMI. Introducing a appropriate non‐singular transformation, $T_{c}=\left[\begin{array}{cc} P_{c}^{-1} & 0\\ 0 & I_{n\times n} \end{array}\right]$ one can easily show

(38) $$W_{c}(P_{c},K|\alpha_{c},\varepsilon_{c})<0\Leftrightarrow T_{c}^{T}W_{c}(P_{c},K|\alpha_{c},\varepsilon_{c})T_{c}<0$$

□

Expanding the elements of the matrix, $W_{c}(P_{c},K|\alpha_{c},\varepsilon_{c})$ and carrying out the operation as mentioned in (38 ) with the introduction of a new variable $\Lambda_{m}=(A+BK+\left(\alpha_{c}/2\right)I_{n\times n})P_{c}^{-1}+P_{c}^{-1}((A+BK+\left(\alpha_{c}/2\right)I_{n\times n}){\;}^{⊺}+\varepsilon_{c}L_{\phi }^{2}I_{n\times n}P_{c}^{-2}$, one can write

(39) $$T_{c}^{T}W_{c}(P_{c},K|\alpha_{c},\varepsilon_{c})T_{c}=\left[\begin{array}{cc} \Lambda_{m} & I_{n\times n}\\ I_{n\times n} & -\varepsilon_{c}I_{n\times n} \end{array}\right]<0$$

Now the term $P_{c}^{-2}$ can be estimated by the following inequality, $P_{c}^{-2}<Q$ . Applying Schur complement lemma in (9 ) to this inequality, one can obtain

(40) $$\left[\begin{array}{cc} Q & P_{c}^{-1}\\ P_{c}^{-1} & I_{n\times n} \end{array}\right]>0$$

Defining new variables $X=P_{c}^{-1}$ and $Y_{c}=KP_{c}^{-1}$, (i) the term $\Lambda_{m}$ in (39 ) is modified as $\Lambda_{m}=\Lambda_{11c}$ as defined in Theorem 1 and (ii) substituting these new variables, $X=P_{c}^{-1}$ and $Y_{c}=KP_{c}^{-1}$ in (40 ), one finally obtains the following LMI condition

(41) $$\left[\begin{array}{cc} \Lambda_{11c} & P_{c}\\ P_{c} & -\varepsilon_{c}I_{n\times n} \end{array}\right]<0\left[\begin{array}{cc} Q & X\\ X & I_{n\times n} \end{array}\right]>0$$

While satisfying (41 ), the controller gain matrix can be calculated as

(42) $$K=Y_{c}X^{-1}$$

For obtaining the optimal and realisable value of controller gain matrix, a minimal size of the convex region (ellipsoid) is computed by reformulating the above theorem as a minimization problem. The result is presented in the form of corollary below.

The trace of $P_{\mathrm{att}r_{c}}^{-1}=X$ is minimised such that the closed‐loop states of (47 ) converges to a smaller attractive ellipsoid of ‘minimal’ size. The mathematical formulation of the statement is described below,

(43) $$\frac{\alpha }{\varepsilon_{c}(\delta^{2}X_{+}^{2}+D_{+}^{2})}\mathrm{tr}P_{c}\to \underset{P_{c}>0,K,\alpha_{c}>0,\varepsilon_{c}>0}{\lim\sup}$$

satisfying,

(44) $$\tilde{W}\left(P_{\mathrm{att}r_{c}},K^{*}|\alpha_{c},\varepsilon_{c}\right)<0$$

and the optimal controller gain matrix, $K^{*}$ is computed as

(45) $$K^{*}=Y{{P_{{\mathrm{attr}}_{c}}}^{*}}^{-1}$$

### 2.4 Design of output feedback control law

The objective of this sub‐section is to design an output feedback control law such that the uncertain system in (6 ) with the Lipschitz condition condition (8 ) and the bounds in (7 ) is robustly asymptotically stable with the estimated states, ${\hat{x}}_{d}(t)$ . The closed‐loop dynamics by substituting the following output feedback control law:

(46) $$u=K{\hat{x}}_{d}(t)$$

is given as

$${\dot{x}}_{d}(t)=(A+\Delta A(t))x_{d}(t)+BK{\hat{x}}_{d}(t)+\phi (x_{d}(t),K{\hat{x}}_{d}(t))$$

Using (11 ), one can rewrite the closed‐loop dynamics as

(47) $${\dot{x}}_{d}(t)=(A+\Delta A(t))x_{d}(t)+BKx_{d}(t)-BKe_{o}(t)+\phi (x_{d}(t),K{\hat{x}}_{d}(t))$$

Now considering the Lyapunov candidate function in (35 ) and differentiating it with respect to time followed by substituting (47 ), one can arrive easily at

(48) $$\begin{array}{rl} {\dot{V}}_{c}\left(x_{d}\left(t\right)\right) & ={\left(\begin{array}{c} x_{d}\left(t\right)\\ \xi \left(t\right) \end{array}\right)}^{T}W_{c}(P_{c},K|\alpha_{c},\varepsilon_{c})\left(\begin{array}{c} x_{d}\left(t\right)\\ \xi \left(t\right) \end{array}\right)\\ & \;+\varepsilon_{c}(\delta^{2}X_{+}^{2}+D_{+}^{2})-\alpha_{c}V_{c}\left(x_{d}\left(t\right)\right)-2PBKe_{o}(t) \end{array}$$

Notice that the above equation is exactly the same as (37 ) except the term ‘ $-2PBKe_{o}(t)$ ’. Since Theorem (4) guarantees the convergence of the state estimation error $e_{o}(t)$, the term ‘ $-2PBKe_{o}(t)$ ’ can be neglected. Hence, the state vector $x_{d}(t)$ converges to an attractive ellipsoid.

## 3 Results & discussion

This section presents the closed‐loop simulation results of the designed controller on the T1D subjects with time‐varying uncertain model parameters and uncertain external meal disturbances such that (i) PGC must not fall below the severe hypoglycemic level ($x_{1}>50\;\mathrm{mg}/\mathrm{dl}$ ), (ii) prolonged post‐prandial hyperglycemia in the presence of external meal disturbance is avoided and (iii) the control signal should be non‐negative.

The optimal values for the controller gain matrix, $K^{*}$ in (45 ) is computed using LMI in (44 ), the computed value is $K^{*}=[0.16-727.41-0.036-3.13]$ . The observer gain matrix $L^{*}$ in (27 ) is obtained by solving LMI (26 ) and the value is $L^{*}=[23.13-2.69\times 10^{-5}-3.03\times 10^{-5}-8.26\times 10^{-5}]{\;}^{T}$.

The numerical values for $X_{+}$, $D_{+}$ and δ in (7 ) are considered according to the physiological plausible range as provided in Table 1. As provided in Table 1 the lower bounds of $x_{1_{d}}$, $x_{2_{d}}$ and $x_{3_{d}}$ are considered to be −80, 0 and −7 since they are the deviated states of physical variables (such as glucose and insulin concentrations) whose lower limits are always non‐negative. The Lipschitz constant is computed by considering the above bounds on the states.

### 3.1 Scenario I: intra‐patient variability and inter‐patient variability for single meal disturbance

*Protocol:* In this scenario, the objective is to investigate the controller's ability to bring PGC to normal value from the hyperglycemic state within t=150min in the presence of a high initial meal disturbance. The initial conditions describing the physiological conditions of the T1D patients are $x_{10}=200\;\mathrm{mg}/\mathrm{dl}$, $x_{20}=0.001\;\mathrm{mi}n^{-1}$ and $x_{30}=7\;\mathrm{mU}/l$.

### 3.2 Scenario II: intra‐patient variability and inter‐patient variability with uncertain meal disturbances

*Protocol:* A simulation scenario of 3 days (4320 min) is carried out here, the parameters of the system (6 ) are randomly chosen from Table 1 during each simulation and they vary sinusoidally during the whole simulation time. The meal intake protocol for the subject is according to Table 2, it repeated for the remaining two days as well. To depict a realistic situation of meal intake by the subjects they are chosen to be highly uncertain in terms of magnitude and timing.

**Table 2:** Meal protocol for 1 day (0–1440 min)

Meals	*d* (0), mg/dl/min	Timing, min
breakfast	[5, 10]	[420, 540]
lunch	[5, 10]	[660, 780]
dinner	[5, 10]	[1140, 1260]

### 3.3 Discussion

**Fig 2:**
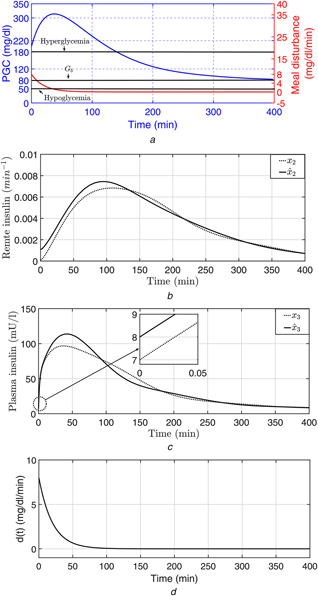
States and the estimated states under intra‐patient variability

**Fig 3:**
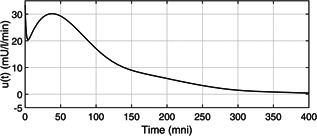
Intravenous insulin infusion rate

**Fig 4:**
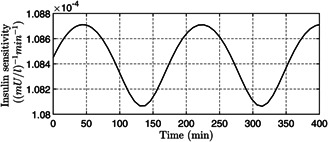
*Time‐varying insulin sensitivity* $\frac{c_{3}}{c_{2}}$

The performance of the robust observer is depicted in Fig. 2 as the the trajectories of the estimated states tracks the original states in the presence of ±30% sinusoidal variations in $c_{i},i=2,\ldots ,5$ . From Fig. 2 *a* one can observe that, the PGC ($x_{1}(t)$ ) is brought to the basal value $G_{b}=80\;\mathrm{mg}/\mathrm{dl}$ successfully by the designed controller in presence of time‐varying uncertainty thus avoiding any instance of hypoglycemia. Further, from Fig. 2 one can notice the effect of the initial external meal disturbance is compensated to avoid post‐prandial hyperglycemia as the sudden overshoot in PGC is brought below 180 mg/dl within t=150min . The control input (insulin infusion rate) determined by the designed controller is depicted in Fig. 3, one can notice that the control signal is non‐negative, there by eliminating any need for auxiliary glucose infusion or glucagon delivery system. Form the above discussion it is quite clear that the proposed controller successfully regulates the PGC within the euglycemic range despite the time‐varying physiological parameters such as insulin sensitivity, $\frac{c_{3}}{c_{2}}$ as depicted in Fig. 4.

**Fig 5:**
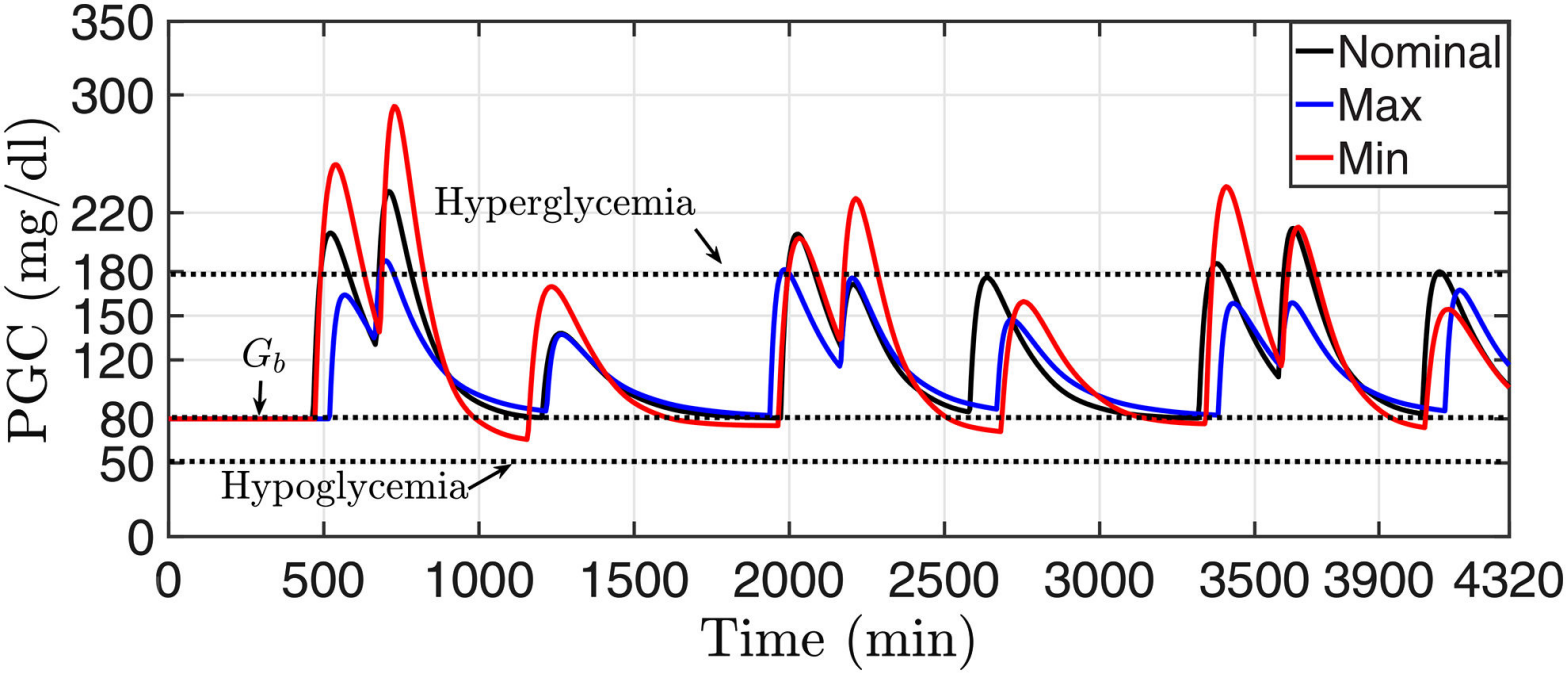
Blood glucose trajectories for virtual T1DM patients

**Fig 6:**
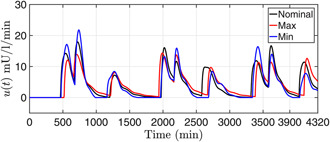
Intravenous insulin infusion rate for virtual T1DM patients

Three virtual T1D patients referred as nominal, maximum and minimum takes three different set of values of $c_{i},i=2,\ldots ,5$ chosen from the uncertainty range specified in Table 1. One can infer from the trajectories presented in Fig. 5 that PGC for these three cases are safely regulated within the euglycemic range (70–180 mg/dl). Due to uncertain meal disturbances, there are certain glucose excursions where the BGC>180 mg/dl. The corresponding control signals (insulin infusion rates) are illustrated in Fig. 6 making it evident that as the PGC approaches the basal value the insulin infusion rate diminishes.

**Fig 7:**
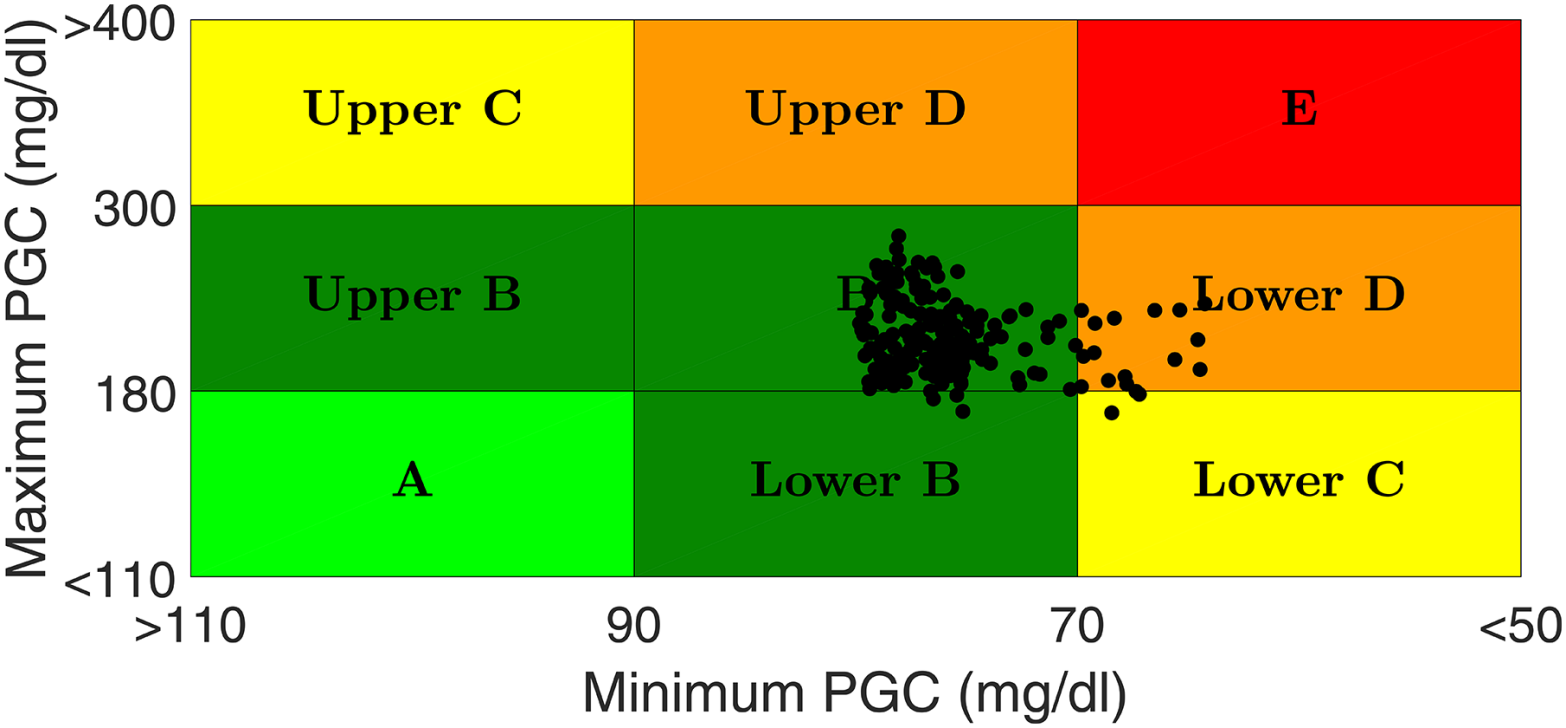
*CVGA for parametric variability of* ±30%

Control variability grid analysis (CVGA) [30 ] is performed to show the efficacy of the proposed robust output feedback control method. For CVGA, 200 random virtual patient parameters are considered for simulation. It is evident from Fig. 7, that 92% of the black dots (closed‐loop simulations) are confined to the Grid *B*, 6% of the dots lie in Grid *Lower D* and 1% are in Grids *Lower B* and *Lower C*, respectively. So, it can be concluded that the proposed control technique completely avoids hypoglycemia (BGC<50 mg/dl) thus validating its effectiveness in dealing with the intra‐patient variability. Further, long‐term implications of post‐prandial hyperglycemia is also significantly reduced by the proposed method.

### 3.4 Comparative study of the proposed control technique with some existing controllers

A comparative analysis of the closed‐loop performance of the proposed AEM observer based controller with single order sliding mode control (SOSMC) [31 ] and higher order sliding mode control (HOSMC) [32 ] is presented in Table 3. As provided in Figure 4 of [31 ], the SOSMC brings the PGC of the T1D patient below 180 mg/dl and the time taken to bring $x_{1}$ below 180 mg/dl, $t_{x_{1<180}}=200\;\min$, and ultimately to the basal value, $G_{b}$ and the time taken to bring $x_{1}$ to $G_{b}$, $t_{x_{1=G_{b}}}=700\;\min$ . As illustrated in Fig. 2, the proposed AEM controller brings the PGC below 180 mg/dl within 120 min which is crucial for avoiding post‐prandial hyperglycemia. Similarly in Figure 3 of [32 ], the HOSMC causes the PGC to fall below 180 mg/dl within 40 min which is very fast response and requires a very high and aggressive control action (insulin infusion rate) as shown in Table 3 with maximum value of control action, $u_{\max}=60\;\mathrm{mU}/l/\min$ . Such aggressive control action can be dangerous due to hypoglycemia if the PGC of the T1D patient is at lower values. As depicted in Fig. 3 the nature of the control action of the AEM controller is more acceptable in terms less aggressiveness and absence of chattering phenomenon in the control signal, that are present in the control signals of Figure 4 of [31 ] and Figure 4 of [32 ]. It is note worthy to mention that the SOSMC and HOSMC has not considered the effect of meal disturbance d(t), unlike the present work as mentioned in Table 3.

**Table 3:** Comparison of AEM, SOSMC FOLTG, HOSMC and STC

Parameters	AEM	SOSMC [31 ]	HOSMC [32 ]
$x_{1_{0}}$, mg/dl	200	300	200
$t_{x_{1<180}}$, min	110	200	40
$t_{x_{1=G_{b}}}$, min	400	700	400
d(t), mg/dl/min	8	—	—
$u_{\max}$, mU/l/min	30	6	60
impulse in	absent	absent	present
control signal	—	—	—
chattering	absent	present	absent
phenomenon	—	—	—

## 4 Conclusion

A simple yet effective robust observer based output feedback control technique based on the attractive ellipsoid method has been proposed for an uncertain Bergman's minimal model. The CVGA plot clearly reveals the effectiveness and reliability of the proposed technique in regulating the glucose concentration and insulin infusion near to normal subjects under parametric uncertainty and random meal intake. Severe hypoglycemic events which are the primary concern of Artificial Pancreas is eliminated successfully. The future scope of the proposed robust observer based control technique based on the attractive ellipsoid method can be the extension of the robust control philosophy to the more complicated subcutaneous T1DM model that finds an immediate application in the Artificial Pancreas System.

## 5 Acknowledgment

The authors thankfully acknowledge the support of Prof. Alexander Poznyak, CINVESTAV‐IPN, Mexico D.F. for his constructive technical inputs towards completing the work.
